# Two-Dimensional UVA Dose Mapping Using a TTC-Pluronic F-127 Hydrogel Dosimeter

**DOI:** 10.3390/ma19132757

**Published:** 2026-06-29

**Authors:** Elżbieta Sąsiadek-Andrzejczak, Marek Kozicki

**Affiliations:** Department of Mechanical Engineering, Informatics and Chemistry of Polymer Materials, Faculty of Textiles and Design, Lodz University of Technology, Żeromskiego 116, 90-543 Lodz, Poland

**Keywords:** hydrogel dosimeter, dose distribution measurements, UV radiation, 2D dosimeter

## Abstract

Monitoring ultraviolet (UV) radiation dose distribution is crucial in many fields, like medicine and materials science, but traditional point-of-care methods limit the ability to fully assess the spatial extent of the irradiated surface. This paper presents the characterisation of a two-dimensional (2D) dosimetry system based on Pluronic F-127 hydrogel matrix doped with 2,3,5-triphenyltetrazolium chloride (TTC) with respect to exposition to UVA radiation. The hydrogel matrix (25% *w*/*w*) provides both high transparency and mechanical stability, while TTC (0.1% *w*/*w*) functions as a colour precursor that undergoes irreversible reduction to form water-insoluble red formazan upon UVA exposure. The insolubility of TTC formazan ensures that the resulting colour changes remain spatially stable within the dosimeter. The study included sample preparation in flat PMMA containers and analysis of the effect of radiation field uniformity in a UVP CL-1000 exposure chamber. It was supported by application of Kodak X-Omat 100 NIF UV Film dosimetry. The actual dose distribution in the chamber was shown to be significantly heterogeneous (CV coefficient of variation of approximately 18%), which emphasises the need for 2D dosimeters for precise validation of irradiation devices. The use of flatbed scanning and dedicated image analysis software allowed obtaining precise 2D dose distribution maps. The dosimeter was characterised in the dose range of 0–5000 mJ/cm^2^, showing a reproducible response (R^2^ = 0.9967). A resolution test was conducted to assess the precision of geometric representation. In the final stage of the study, the suitability of the developed dosimetry system was verified under conditions simulating heterogeneous UV radiation dose distribution using patterns printed with Computer-to-Film (CtF) technology. The results showed that optical effects in printed films significantly affect UV transmission, limiting accurate dose recording for black coverage above approximately 40–50%. The results obtained confirm that the TTC-Pluronic F-127 system is an effective, simple and low-cost tool for 2D monitoring of UVA radiation, with potential applications in cosmetology, dermatology, and material ageing tests.

## 1. Introduction

UV radiation is essential for the proper development of ecosystems and has a positive impact on human health; however, excess UV radiation causes serious damage. Regardless of its artificial or natural origin, UV radiation can be divided into three types: UVA (315–400 nm), UVB (280–315 nm), and UVC (100–280 nm), which differ in wavelength and interaction. Overexposure to UV radiation can cause skin irritation and burns and, in the long term, lead to excessive and premature ageing, eye damage, immune dysfunction, DNA damage and degradation, and cancer [1,2,3,4,5,6]. Although many professions are exposed to UV radiation, such as construction workers, medical and aesthetic medicine professionals, nail technicians, and welders, according to the International Commission on Non-ionising Radiation Protection and the World Health Organization, there are no clear recommendations regarding the use of dosimeters for monitoring radiation in the 100–400 nm range [7,8,9,10]. In the case of direct exposure to sunlight, it is recommended to use the UV index (UVI), which is a standard measure of the intensity of UV rays reaching the Earth’s surface depending on location, season, and time. The UVI index is primarily used to determine the risk of sunburn and eye damage, which also translates into the recommendation of wearing sunglasses with certified filters and applying sun protection filters (SPF). In the case of contact with artificial sources of UV radiation, it is recommended to use protective clothing with specific design parameters and physical and chemical finishes that provide a high ultraviolet protection factor (UPF), which measures the ratio of the dose of solar radiation causing redness on skin protected by textiles to the dose of solar radiation causing the same effect on skin without any protection [11,12,13,14]. In the field of UV radiation measurement, the most frequently discussed methods include electronic meters, photodiodes, sensors based on inorganic materials, photostimulated and thermoluminescent materials, liquid crystals, biological dosimeters, and dye solutions [15,16,17,18], which are typically used as point-based (0D) measurement methods, significantly limiting the ability to assess the dose distribution. The development of gel dosimeters, which enable dose measurements in 2D and 3D formats, has been a particularly popular approach.

Among the many developed solutions, interesting dosimeters appear to be those that change colour or bleach upon absorbing a specific dose of UV radiation. These can be found in polymer tablets, flat polymer films, layers printed on the surface of paper or textile products, or volume-modified fibres. Noteworthy are those made of poly(ethylene oxide)-block-poly(propylene oxide)-block-poly(ethylene oxide (Pluronic F-127) hydrogel, which are completely transparent before irradiation and exhibit a visible, intense colour change after irradiation [19,20,21]. The advantage of hydrogel matrices with a low light-scattering coefficient is that they do not contribute to anomalies associated with the reading of colour changes after irradiation and, consequently, the collection of information on the dose and its distribution. It is also important that the colour change in such dosimeters is stable over time and visible, with its intensity increasing with increasing absorbed radiation. The potential of the Pluronic F-127 matrix has been successfully validated in ionising radiation dosimetry. The Fricke-XO-Pluronic F-127 system, which utilises ferrous ions and xylenol orange, has been shown to be successfully used as a 2D dosimeter for radiation isocentre verification in medical linear accelerators. These studies demonstrate that Pluronic F-127-based hydrogels provide the mechanical and optical stability necessary for precise dose distribution measurements, paving the way for their adaptation to other radiation ranges, including UV. Previous studies have also shown that the exemplary hydrogel systems with TTC, LMG, and LCV are suitable for monitoring UVA and UVB radiation, and the dose sensitivity of the dosimeters is 3.60 ± 0.12, 42.85 ± 1.53, and −1.32 ± 0.11 cm^2^/J, respectively [20,21]. However, the scope of the work performed did not include the use of Pluronic F-127 hydrogels doped with UV-sensitive colour precursors for measuring radiation dose distribution on a 2D surface but focused primarily on dose-response characterization and sensitivity assessment. The studies provided fundamental information on their optical and dosimetric properties. In the context of UV radiation detection, TTC is a well-known radiosensitive colour precursor. Upon exposure to radiation, it irreversibly reduces to a coloured formazan, allowing for visual and spectrophotometric assessment of the absorbed dose. The photoreduction of TTC to formazan proceeds via electron transfer processes initiated by radiation-induced reactive species, leading to the formation of a stable, water-insoluble coloured product. This mechanism may be influenced by the local chemical environment, including oxygen concentration, the presence of reducing intermediates, and the properties of the surrounding matrix. In particular, dissolved oxygen can act as a competing electron acceptor, potentially affecting the efficiency of formazan formation. Additionally, the hydrogel matrix may influence reaction kinetics by modifying diffusion processes and the availability of reactive species. It should be emphasised, however, that previous research on the use of a TTC system in combination with a polymer matrix has focused primarily on applying a thin layer to textile substrates via screen printing or in the form of simple capsules for underwater environmental measurements. Therefore, there is a research gap regarding the characterisation and optimisation of TTC-Pluronic F-127 hydrogel systems dedicated exclusively to UV radiation dose and dose distribution measurement, analogous to systems used in radiotherapy.

The aim of this work concerns the analysis of a hydrogel dosimetry system with the following requirements: (i) the radiosensitive component of the dosimeter must respond to UV radiation; (ii) the dosimeter must have a transparent gel matrix with low light scattering; (iii) the dosimeter’s response to the absorbed dose must be visible as a colour change of the radiosensitive component; (iv) the dosimeter must have a flat 2D form to be imageable using a flat-bed scanner; (v) the hydrogel system must not interact with the light from the scanner; and (vi) the dosimeter must be stable before and after irradiation. Based on previous research on radiochromic dosimeters, the decision was made to use a Pluronic F-127 hydrogel matrix with TTC as a UV-sensitive compound. The scope of work included: (i) preparation of TTC-Pluronic F-127 dosimeters in flat containers suitable for imaging with a flat-bed scanner; (ii) irradiation and imaging of samples with a flat-bed scanner; (iii) calibration of the dosimeters after irradiation, along with determining the equation describing this relationship; and (iv) processing of the data obtained from the scanned images using 2D computer analysis software, including the preparation of UV dose distribution maps. This paper presents another potential use of such radiochromic hydrogels as a complement to systems for verifying the uniformity of the illumination field in UV ageing chambers, for monitoring lamps used in cosmetology and dermatology, and for developing a new approach to preparing dosimeters for monitoring the distribution of the 2D UV dose generated by natural and artificial light sources. Although TTC-based systems, including Pluronic F-127 hydrogels, have previously been investigated for UV detection, these studies have primarily focused on point measurements, simple geometries, or qualitative assessments of colour change. This work expands on these approaches by presenting a fully 2D dosimetry system that allows for quantitative mapping of UVA dose distributions using image-based analysis. The main innovation of this study is the development of a reproducible methodology for preparing flat hydrogel dosimeters suitable for dose sensing, combined with a calibration and data processing system that allows for spatial reconstruction of the dose distribution in 2D. Furthermore, the system is evaluated under real-world irradiation conditions, including highly inhomogeneous fields and irradiation with CtF film templates, which reveals significant practical limitations related to UV transmission and optical artefacts of the developed dosimeter.

## 2. Materials and Methods

### 2.1. Preparation of Samples

**Figure 1:**
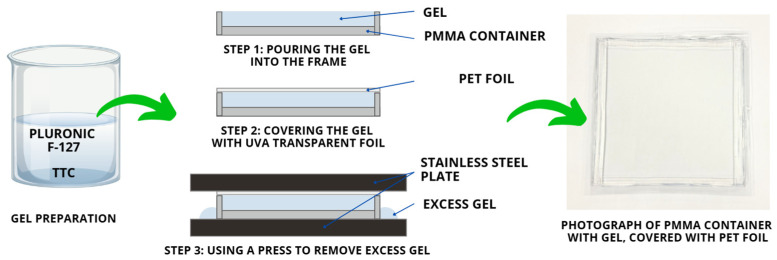
Scheme of Pluronic-F127-TTC sample preparation in cuboidal PMMA containers.

The radiochromic gel composition was prepared from redistilled water, poly(ethylene oxide)-block-poly(propylene oxide)-block-poly(ethylene oxide) (Pluronic F-127, Sigma-Aldrich, Saint Louis, MO, USA; Mw = 12,600 g/mol) as a gel matrix and 2,3,5-triphenyltetrazolium chloride (TTC, Sigma-Aldrich, Saint Louis, MI, USA; M = 334.81 g/mol) as a radiation-sensitive colour precursor. The gel made from Pluronic F-127 provides a colourless, highly transparent matrix that does not change colour after UV irradiation. An additional advantage is the approval of Pluronic F-127 by the US Food and Drug Administration (FDA) as a non-toxic copolymer, which is not harmful to humans or the environment in the event of direct contact with the compound or during its disposal [22]. The TTC was dissolved in distilled water at room temperature using a magnetic stirrer for 24 h before being mixed with the gel matrix. This solution was then cooled to 5 °C over 1 h and mixed into a cool Pluronic F-127 aqueous solution (33% *w*/*w*) prepared 72 h earlier. It should be emphasised that depending on the copolymer concentration and ambient temperature, Pluronic F-127 can be in the form of an aqueous solution or a physical gel. For example, a 25% aqueous solution of Pluronic F-127 at 4 °C is easily mixed with an aqueous solution of a UV-sensitive colour precursor, while at a temperature of 19–85 °C [23], it will form a physical gel. The final concentration of Pluronic F-127 in the gel was 25% *w*/*w*, and the TTC concentration was 0.1% *w*/*w*. Gel samples were then prepared for ultraviolet irradiation. Cuboidal poly(methyl methacrylate) (PMMA) containers measuring 12 cm × 12 cm × 0.3 cm were used for this purpose. During sample preparation, the container was placed on a levelled stainless-steel plate. To better visualise the sample production process, Figure 1 presents a flowchart of the sample production process, which included three steps: (i) pouring the gel into containers, ensuring that the gel filled the prepared frame to the brim, (ii) covering the gel with a polyethylene terephthalate foil (PET; 0.10 mm thick; UVA-transparent; Folex AG, Seewen, Switzerland), and (iii) removing the excess gel that was pushed out of the container after applying a stainless-steel pressure plate. The sample remained between the plates at room temperature 23 °C, for 2 h until it transformed into a physical gel. After this time, the pressure plates were removed, and the sample was ready for irradiation. The described sample preparation method guaranteed reproducible production. In particular, the use of a rigid PMMA frame and controlled compression ensured a uniform and repeatable gel thickness across the entire surface of all samples. It is known that gel parameters, such as thickness, can affect the dosimeter’s response to radiation. In the presented work, no other sample preparation methods were used, nor were gel thickness variations used. Therefore, the influence of structural parameters, particularly gel thickness, on the dosimeter’s response to UV radiation was not investigated in this study. However, it should be noted that maintaining a constant and well-defined thickness is essential for ensuring reproducible optical response and spatial accuracy in 2D dosimetry systems.

### 2.2. Sample Irradiation

All Pluronic-F-127-TTC gel samples in PMMA containers were irradiated in a UV crosslinker chamber CL-1000 (UVP, Toronto, ON, Canada) with UVA radiation (range 315–400 nm; peak at 365 nm; five 8 W lamps; type F8T5 Blacklight; Hitachi, Tokyo, Japan). The desired UV dose (mJ/cm^2^) was automatically delivered using a built-in detector and chamber control system. To verify the dose emitted by the crosslinker, an external electronic UV meter Sentry ST510 was used (sensor measurement spectrum 320–380 nm; sensor calibration point 365 nm; sampling rate 3×/s; accuracy (23 °C ± 5 °C) ±4% ± 1 count for measurements >1 mW/cm2; Sentry Optoelectronics Corp., Taipei, Taiwan). For this purpose, 20 radiation measurements were performed in the crosslinker chamber. The first series recorded a dose of 500 mJ/cm^2^ and an emission time of 88 ± 0.3 s. In the second series, the Sentry ST510 detector was covered with a 0.10 mm thick PET foil, and 20 UV radiation measurements were again performed for the same dose. Comparing the measurements from the first and second series, the emitted dose was determined to be 473.8 ± 22.7 mJ/cm^2^ and 373.9 ± 18.1 mJ/cm^2^ for measurements without and with the PET foil cover, respectively. Based on this, the dose absorbed by the Pluronic F-127-TTC samples was calculated to be 21% lower than the dose set on the crosslinker. It should be emphasised that all samples used in the presented work were covered with PET foil. Therefore, to avoid additional calculations, the UVA radiation doses (mJ/cm^2^) indicated in this paper are considered the dose emitted by the CL-1000 crosslinker.

**Figure 2:**
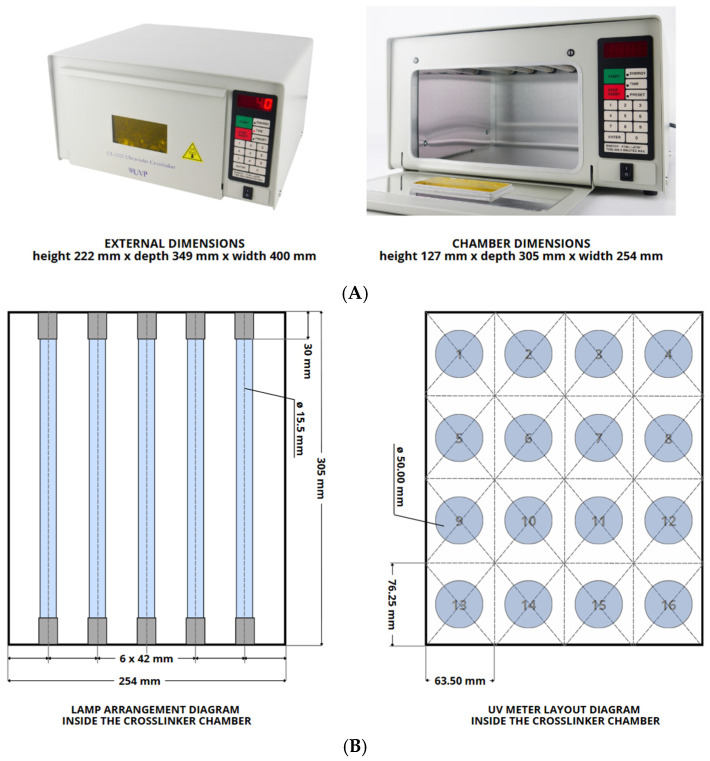
An external and internal view of the Analytik Jena UVP CL-1000 crosslinker chamber with dimensions (**A**) and a diagram of the lamp arrangement inside the device with the division adopted when performing measurements with the Sentry ST510 external UV meter (**B**). Numbers in B correspond to the positions of samples.

The UV Sentry ST510 m was also used to assess the uniformity of the radiation field of the CL-1000 crosslinker chamber. To explain the method of preparing measurements using a UV meter, Figure 2A presents the external and internal view of the UVP CL-1000 crosslinker chamber, including the arrangement of the UVA lamps and the working area geometry. Figure 2B illustrates the adopted measurement grid and the positioning of the UV meter detector within the chamber. The chamber base was divided into sixteen fields (4 rows × 4 columns with dimensions of 76.25 mm × 63.50 mm), and measurements were performed at the centre of each field, defined by the intersection of the diagonals, ensuring consistent and reproducible detector positioning. Before measuring UVA radiation for the specified doses, the crosslinker was set to emit a dose of 1 J/cm^2^ to warm up the UV lamps and reduce measurement errors related to their operating temperature. A detector was then placed in the centre of the measurement field, and the radiation intensity was recorded for the specified UVA doses of 100, 500, and 1000 mJ/cm^2^ for 18, 88, and 195 s, respectively. The intensity measurements were converted into a radiation dose as the product of the intensity measurement and the emission time of the set dose.

The uniformity of each field was quantified by calculating the minimum, maximum, and mean dose and the coefficient of variation (CV), where CV is defined as the ratio of the standard deviation to the mean UV dose. Mapping was performed for three radiation doses of 100, 500, and 1000 mJ/cm^2^. Additionally, Kodak X-Omat 100 NIF UV film (Rochester, NY, USA) was used to assess the uniformity of the radiation field of the CL-1000 crosslinker chamber. In the first stage, film samples (30 mm × 30 mm) were irradiated with UVA radiation at doses up to 600 mJ/cm^2^, and a calibration equation was determined. Next, two films (210 mm × 297 mm) were irradiated with doses of 15 and 30 mJ/cm^2^ and analysed as described in Section 2.3.

For inhomogeneous irradiation, black, opaque templates were printed using the Computer to Film (CtF) printing method (Heidelberg Herkules Pro; round raster; raster ruling 7000; Heidelberger Druckmaschinen AG; Gutenbergring, Germany). To determine the change in UVA radiation dose through the CtF film, UV radiation passing through the CtF film was also measured (set dose was 500 mJ/cm^2^). Based on 20 measurements, the dose measured with the Sentry ST510 metre was 377.4 ± 14.7 mJ/cm^2^, which is approximately 20.3% of the emitted dose. Therefore, the PET foil and CtF film used in this study were considered similar. It is important to emphasize that UV dose measurements conducted without CtF film confirmed that the TTC-Pluronic F-127 dosimeter exhibited a repeatable dose response, indicating that the distortions observed in the CtF experiments are primarily due to optical effects within the film layer and not to intrinsic dosimeter limitations. Furthermore, the UV doses reported in this study correspond to the nominal (emitted) dose values set on the CL-1000 crosslinker, which constitute the controlled experimental parameter. Although attenuation of UV radiation by intermediate materials such as PET foil and CtF films was observed, the real absorbed dose depends on multiple factors, including material properties and spatial variations in the radiation field, and therefore cannot be uniquely defined under all conditions. Consequently, the nominal emitted dose was used consistently throughout the study to ensure comparability and reproducibility of the results.

### 2.3. 2D Scanning and Data Processing

All samples were scanned using a flatbed scanner, the Epson Perfection V750 Pro (Seiko Epson Corp., Suwa, Japan). Epson Scan software version 3.9.2.2 (Seiko Epson Corp., Suwa, Japan) was used for scanning, with all image enhancement options disabled, such as unsharp mask, shadow correction, and tonal correction. Based on previous research, samples were scanned at 75 dpi and saved in the BMP file format. All samples were scanned approximately 15 min after UVA exposure. The BMP images obtained after scanning the dosimeters were processed using the ImageJ software (version 1.53t, National Institutes of Health, Bethesda, MD, USA) and the polyGeVero^®^-CT software package (v.1.3, GeVero Co., Łódź, Poland) [24] and prepared for further analysis, including (i) filtering the images after scanning the dosimeter; (ii) calibration of the dosimeter for the UVA radiation dose; (iii) conversion of the obtained results into the UVA radiation dose distribution; and (iv) creation of 2D and 3D maps with the UVA radiation dose distribution.

## 3. Results and Discussion

### 3.1. Uniformity of the UV Radiation Field in the Crosslinker Chamber

**Figure 3:**
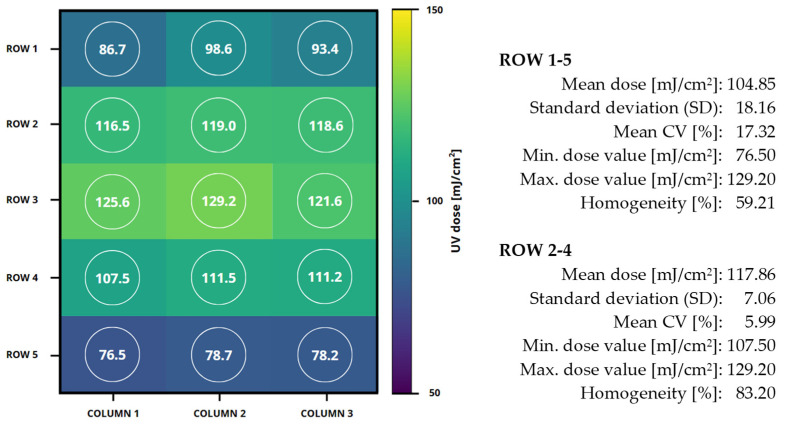
Basic inhomogeneity characteristics of the Analytik Jena UVP CL-3000 crosslinker chamber—own drawing based on Figure 2 in [25].

The homogeneity (%, calculated as Homogeneity = 100–CV, where coefficient of variation CV% = μ/σ × 100% (σ—standard deviation and μ—mean value of radiation dose)) of the UV radiation dose distribution in the sample exposure chamber is one of the key parameters influencing the repeatability and comparability of experimental results. In laboratory practice, it is often assumed that irradiation chambers provide a uniform radiation field, but empirical data from manufacturers indicate that most commercially available devices do not guarantee a uniform UV radiation field. For most crosslinker models, manufacturers do not provide information on the radiation field uniformity. However, for CL-3000 series UVP devices (not used in the present work), this problem is described in detail [25]. The manufacturer’s data indicates that the UV radiation distribution for a new device is non-uniform, with a coefficient of variation (CV) of 17.3% across the entire exposure area (Figure 3). In the central zone of the chamber, where direct radiation and multiple reflections accumulate, the CV drops to 6.0%, confirming greater dose uniformity. These data indicate that the radiation field in the chamber is significantly non-homogeneous and that achieving repeatable UV dose values strongly depends on sample position within the chamber. Absolute UV dose measurements revealed a reduction in exposure (>20% below the target 100 mJ/cm^2^) in the front and left zones of the crosslinker chamber. This discrepancy is attributed to edge effects resulting from geometric distortions, reduced secondary reflections, and energy losses at unfavourable light emission angles from linear UV sources. In multi-lamp systems, the inhomogeneous UV distribution results from: (i) directional and geometrically non-monochromatic emission of low-pressure lamps, (ii) variable reflector efficiency, and (iii) radiation attenuation over distance. Such spatial inhomogeneity directly affects experimental outcomes and may generate systematic errors, distort dose–response curves, and hinder reproducibility. To mitigate these effects, the manufacturer recommends placing samples in the central area, applying a compensating dose on the peripheries, and using an external radiometer for field mapping. Given the structural and technical similarities between CL-1000 and CL-3000 crosslinkers, these observations are expected to be consistent for both models. Based on measurements with an external electronic UV meter Sentry ST510, a basic characterisation of the CL-1000 chamber was prepared, along with two-dimensional dose distribution maps with uniformity analysis at target doses of 100, 500, and 1000 mJ/cm^2^ (Table 1, Table 2 and Table 3). The UV radiation distribution in the CL-1000 chamber showed significant discrepancies between the set and measured with an external UV meter dose. A systematic underestimation of the measured dose was observed for all dose levels. Furthermore, in the rear and front wall areas of the chamber (rows 1 and 4), the measured dose was at least 40% lower than the set dose. Analysis of the 2D radiation doses revealed significant non-uniformity across the working surface of the crosslinker chamber (≈60%), with a clearly defined higher-dose region in the central area (rows 2–3), where the homogeneity reached approximately 93%. Despite high spatial variability and the lack of lamp replacement for over three years, the crosslinker demonstrated high measurement repeatability. A comparison of the emission characteristics of the CL-1000 and CL-3000 chambers revealed differences in absolute dose values, while maintaining similar levels of spatial homogeneity.

**Figure 4:**
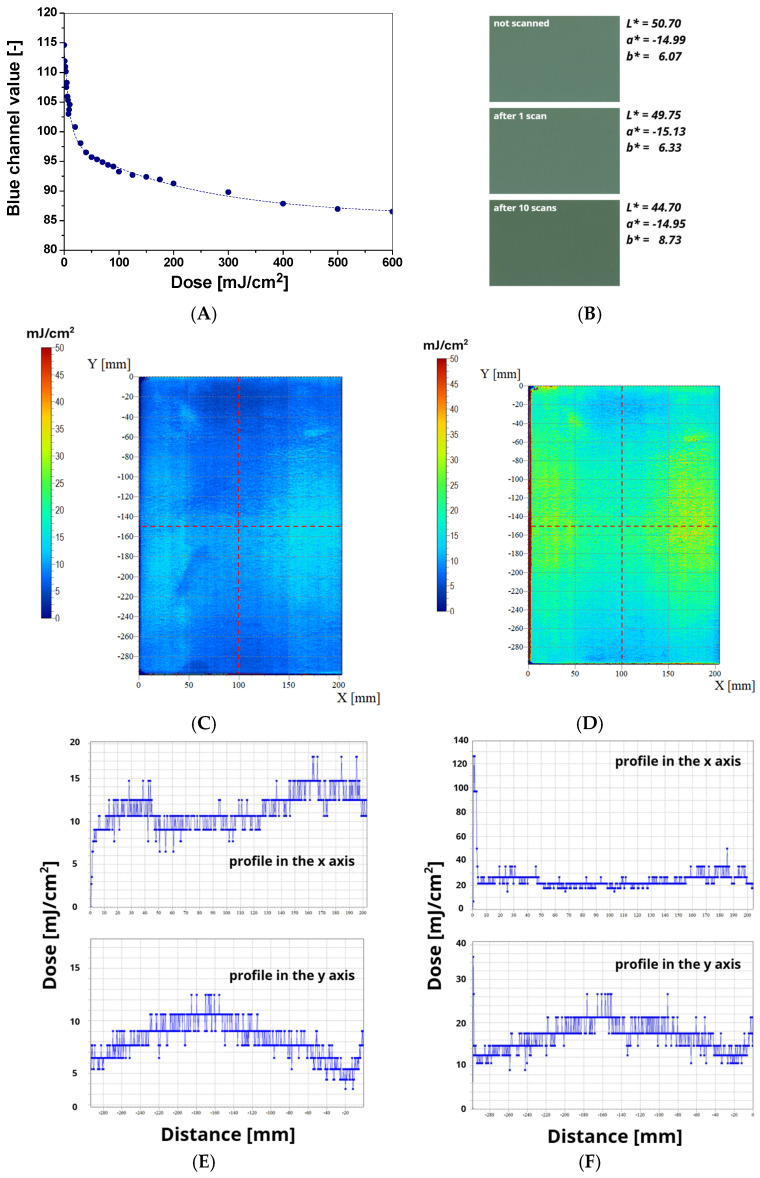
Evaluation of the UVA radiation dose distribution inside the CL-1000 crosslinker chamber using Kodak dosimetric film. (**A**) Calibration relationship for doses up to 600 mJ/cm^2^. (**B**) Colour change of Kodak film samples depending on the number of scans; L*a*b* colour measurements were performed using a 3nh ST60 reflectance spectrophotometer (8 mm aperture, D65 illumination, 10° standard observer, specular component included (SCI mode); Threenh Technology Co., Ltd., Guangzhou, China). The colour differences were evaluated in the CIE L*a*b* colour space, where L* represents lightness, and a* and b* correspond to the green–red and blue–yellow colour components, respectively. (**C**,**D**) 2D map of the dose distribution on the surface of Kodak film irradiated with doses of 15 mJ/cm^2^ and 30 mJ/cm^2^. (**E**,**F**) Dose relationship as a function of distance along the x- and y-axes for profiles marked with red dashed lines on (**C**,**D**).

While the CL-1000 model exhibited a systematic tendency to underestimate the set dose (mean dose 77.81 mJ/cm^2^) for a set dose of 100 mJ/cm^2^, the CL-3000 model showed a slight emission overshoot, reaching a mean dose of 104.85 mJ/cm^2^ (Figure 3). Both devices exhibited similar levels of homogeneity throughout the chamber (CV = 17–18%). Differences were observed in the central regions, where the CL-1000 model demonstrated higher stability in rows 2–3 (CV = 2.46%; homogeneity = 93.68%) compared to the CL-3000 model in rows 2–4 (CV = 5.99%; homogeneity = 83.20%). Consequently, the CL-3000 system provides better dose accuracy, whereas the CL-1000 chamber ensures higher spatial uniformity within the working area. For the CL-1000 chamber, the central zone showed the highest uniformity for all investigated doses (100, 500, and 1000 mJ/cm^2^); therefore, only the fields R2C2, R2C3, R3C2, and R3C3 were used in further experiments. To further verify the radiation distribution in this area, dose measurements were performed using Kodak X-Omat 100 NIF UV film. Film samples were irradiated with UVA doses up to 600 mJ/cm^2^, scanned with a flatbed scanner, and analysed in ImageJ v. 1.54. Based on the change in colour intensity in the blue channel of the RGB colour system, a calibration equation was determined (Figure 4A), described by a second-order exponential decay function: Blue channel = 12.652 × exp(−Dose/224.281) + 15.489 × exp(−Dose/9.605) + 85.795 (Pearson coefficient = 0.9968; R^2^ = 0.9926). Dosimetric films are highly sensitive to mechanical damage and light exposure; therefore, proper calibration and controlled readout conditions are essential. It was observed that Kodak film undergoes colour changes during scanning. Based on colour measurements using a reflectance spectrophotometer under controlled and repeatable condition settings, a sample scanned once was 1.87% darker, while a sample scanned ten times showed an 11.83% difference in brightness (Figure 4B). The intensity of the colour change in such films is undoubtedly influenced by the lamp’s power and scanning speed, which depends on the selected resolution. Therefore, all scans were performed once at 75 dpi. To map the dose distribution, two Kodak films (210 mm × 297 mm) were irradiated at 15 and 30 mJ/cm^2^, selected within the linear response range. The scanned images were processed using polyGeVero^®^-CT software (Section 2.3). After assigning the calibration equation, UVA dose distribution maps were generated and are shown in Figure 4C,D. Additionally, *X*- and *Y*-axis profiles were determined for both samples to describe the nature of changes after irradiation (Figure 4E,F). The results confirm non-uniform dose distribution, with local fluctuations visible both in the central region and near the edges. Minor discrepancies between set and measured doses may result from previously described factors, including chamber geometry, angular sensitivity of the film, and lamp ageing. Additionally, film handling (e.g., bending or cutting) may introduce distortions, although experimental precautions were taken. The high dose values observed in the X profile (Figure 4F) are attributed to a cropping artefact and were excluded from the analysis of dose uniformity. In summary, the obtained results indicate non-uniformity of the UV radiation distribution in the crosslinker chamber, which is a characteristic feature of this type of source. The device manufacturer confirms the occurrence of edge effects and recommends placing samples in the central part of the irradiation field, where dose variability is lowest. When working outside the central area, the use of an external radiometer and, if necessary, increasing the set dose are recommended to compensate for these effects [25]. While these recommendations may be sufficient for point-based or small one-dimensional samples, their application becomes problematic for larger samples, such as the 2D dosimeters (12 cm × 12 cm × 0.3 cm) analyzed in this work. In such cases, spatial differences in radiation intensity extend over the entire sample surface, limiting the effectiveness of simple dose compensation or local calibration. Ensuring repeatability would also require strict and consistent sample positioning, and any change in sample size would necessitate modification of the irradiation procedure. These limitations highlight the restricted applicability of standard calibration approaches and the need for a more advanced approach to UV field uniformity control.

### 3.2. UV Dose-Response of Samples

**Figure 5:**
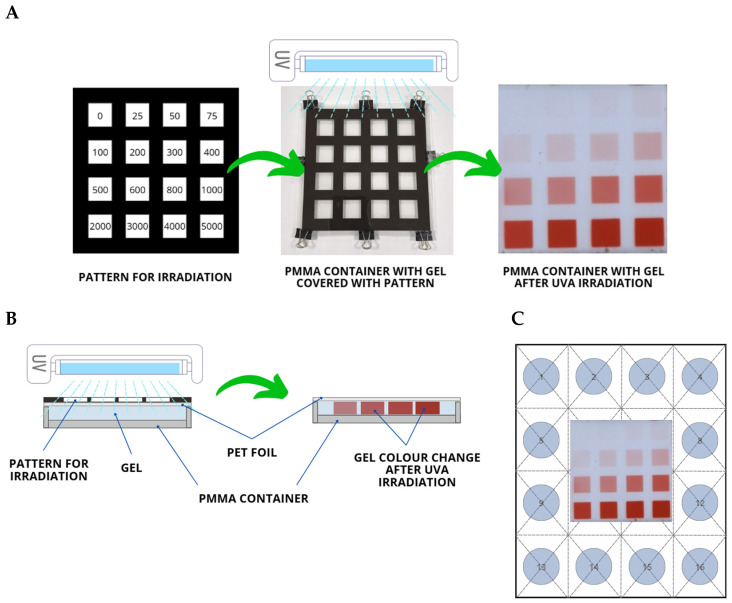
A diagram of the preparation of Pluronic-F127-TTC gel samples for UVA irradiation in the dose range of 0–5000 mJ/cm^2^ using a pattern printed on PET foil using the CtF film method (**A**), a cross-section of the sample showing the effect after irradiation (**B**) and placing the sample inside the crosslinker chamber (**C**).

**Figure 6:**
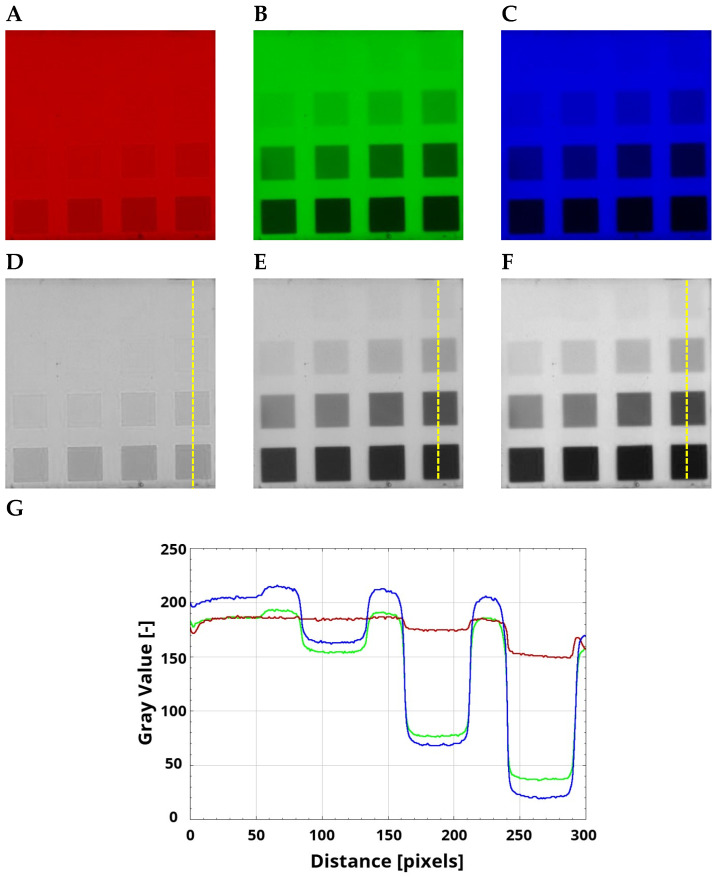
Analysis of colour changes in the RGB channels: red (**A**), green (**B**), blue (**C**), and a map of colour intensity changes in the red (**D**), green (**E**), and blue (**F**) channels on a grey value scale. Comparison of the response after UV irradiation (**G**) was performed for profiles perpendicular to doses of 75, 400, 1000, and 5000 mJ/cm^2^, indicated by the yellow dashed line in images (**D**,**E**,**F**).

The sample prepared according to Section 2.1 was irradiated with UVA radiation in the dose range of 0–5000 mJ/cm^2^. A simple template made of black opaque paper was used for irradiation, as shown in Figure 5A. As a result of irradiation, coloured square areas with dimensions of 1.8 mm × 1.8 mm were formed (Figure 5B). It should be emphasised that in the irradiated areas, the gel did not lose its high transparency, and the colour developed evenly over the entire irradiated surface, becoming more intense with increasing absorbed UV radiation dose. Each dose was emitted once, ensuring irradiation of only one area. To ensure the highest uniformity of UV radiation, the sample was placed in the central part of the crosslinker chamber (R2C2, R2C3, R3C2, R3C3) (Figure 5C). The sample was not touched or moved during irradiation. Approximately 15 min after irradiation, the samples were scanned using an Epson flatbed scanner. As described in Section 2.3, scanning was performed at 75 dpi resolution, and the resulting image was saved as a BMP file, which was then analysed in the ImageJ graphics programme. Analysis of the distribution of colour components in the RGB space revealed differences in the sensitivity of individual channels to the UV radiation dose. Based on the images decomposed into components (Figure 6A–C), corresponding colour change intensity maps were generated within the selected colour channel and presented as grey value (values from 0 to 255) optical density profiles (Figure 6D–F). A profile was then determined along the axis perpendicular to the doses of 75, 400, 1000, and 5000 mJ/cm^2^, as indicated by the yellow dashed line, and the relationship between the optical density change and the pixel distance of the image was determined (Figure 6G).

**Figure 7:**
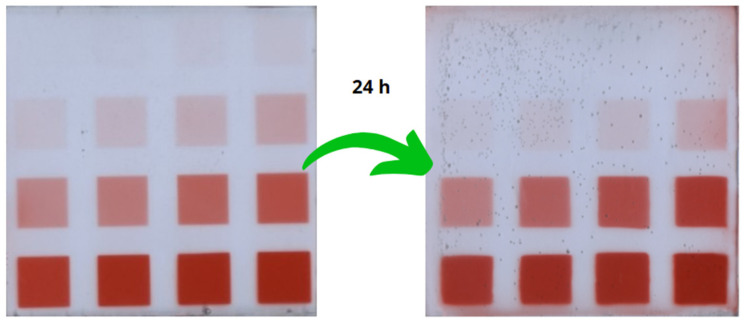
Photograph of the TTC-Pluronic F-127 sample immediately after preparation and UVA irradiation at doses of 0–5000 mJ/cm^2^, immediately after preparation in a PMMA frame, and after 24 h with visible air bubbles. The sample was stored at 22 °C.

Comparing the obtained profiles, the red channel is characterised by the lowest signal dynamics, showing little change in grey levels over the tested dose range. The green channel shows a moderate response, while the blue channel shows the strongest response, especially at higher doses. At the same time, the blue channel also shows a change in the grey value of approximately 5% for unirradiated areas, which were covered with a paper template during irradiation, which may indicate partial transmission of UVA radiation. Therefore, the unirradiated channel was selected for further analysis. A crucial factor in determining the calibration equation is the time from sample preparation to irradiation, and imaging by scanning. After the preparation of TTC-Pluronic F-127 hydrogel, bubble formation was observed within 24 h at room temperature 22 °C (Figure 7). The phenomenon of air bubble formation in aqueous solutions after gelation can be explained by analysing the physicochemical properties of Pluronic F-127 described in the literature [26,27,28,29,30]. Pluronic F-127 is an amphiphilic block copolymer (PEO-PPO-PEO) that exhibits strong surface-active properties and is used, among others, in foam stabilisation and emulsification processes. The gelation mechanism of a 25% solution at room temperature (above the gelation point of approximately 14 °C) involves a sol–gel phase transition from an isotropic micellar structure to an ordered cubic phase. This process is driven by the hydrophobic effect and involves rapid dehydration of polymer blocks during micelle formation [26,27].

An increase in temperature initially causes dehydration of the PPO blocks, which leads to the formation of the micelle core, followed by dehydration of the PEO blocks in the micelle shell. This process alters the local solubility of gases in the aqueous phase, as the water released from the polymer shells loses the ability to retain dissolved air, thereby promoting air nucleation in the form of microbubbles [26]. The next stage is the stabilisation of the bubbles by high viscosity and gel structure. The formation of ordered cubic liquid crystalline structures is associated with a rapid, even 100-fold, increase in solution viscosity and the development of a high elastic modulus. Such a change in rheological parameters immobilises the formed bubbles within the network, creating a so-called kinetic trap. Furthermore, the structure of the Pluronic F-127 hydrogel is not homogeneous. Studies demonstrate the coexistence of areas with different packing densities, so-called large meshes and smaller pores. This heterogeneity allows for the slow, hours-long aggregation of microbubbles trapped within the structure. As a result, the bubbles become visible to the naked eye only after the system stabilises, typically 24 h after gelation, even though the starting solution appeared clear during sample preparation [27,28,29]. It should be emphasised that all irradiation and scanning procedures in this study were performed before visible bubbles occurred, ensuring optical homogeneity of the samples during dose readout. Therefore, the analysis of the dosimeter’s dose response is not significantly affected by bubble-related artefacts. Quantitative assessment of the effect of bubble formation on dose measurement accuracy would require changes in hydrogel preparation procedures and controlled variation of the gel structure, such as its thickness, which is beyond the scope of the presented study results.

Limiting the formation of air bubbles in Pluronic F-127-based hydrogels requires strict control of thermodynamic and rheological parameters during the solution preparation process. Maintaining a low temperature during vacuum degassing or hydrogel centrifugation below the gelation point, i.e., below 14 °C at a polymer concentration of 25% (*w*/*w*), would be effective. To allow air bubbles to float freely to the surface, the solution must be in an isotropic liquid state, as low viscosity at temperatures close to 0–4 °C is crucial. Furthermore, after gelation, Pluronic F-127 hydrogels are characterised by a high elastic modulus. The high stiffness of the crystalline structure makes mechanical removal of bubbles after gelation impossible [26,28]. Intense mechanical mixing should also be avoided, and the rate of the sol-gel phase transition should be controlled, as rapid heating can promote the entrapment of air microbubbles within the rapidly forming micellar network. For example, for a 25% (*w*/*w*) solution at 37 °C, the gelation time is approximately 5 min. All of the above issues have a significant impact on long-term evaluation of the dosimeter’s stability and the reliable ability to assess UV radiation dose response. In this study, all measurements were conducted within a short and controlled time window after the irradiation process due to material and technological limitations. Evaluation of the long-term stability of the developed TTC-Pluronic F-127 dosimeter requires further optimisation of the hydrogel preparation procedure to prevent bubble formation after gelation. This topic may constitute a separate research topic in further work on optimising the proposed dosimeter.

**Figure 8:**
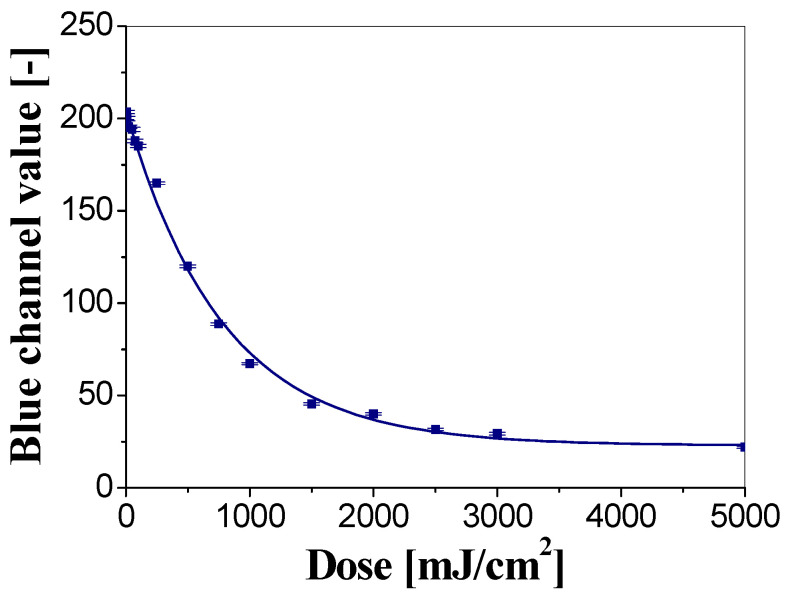
Calibration relations for the blue channel of the RGB colour model after irradiation of samples with UVA radiation in the dose range of 0–5000 mJ/cm^2^.

In the next step, a calibration equation was determined as a function of UVA dose based on the relationship obtained for changes in the grey value scale for the blue channel of the colour space (Figure 8). The obtained calibration curve showed a nonlinear character, described by a first-order exponential decay function, as follows: Blue channel = 22.907 + 180.92 × exp(-Dose/780.573) (Pearson coefficient = 0.9984, R^2^ = 0.9967). It should be noted that the calibration curve was determined based on a single dosimeter sample. The analysis utilised a large number of data points extracted from the scanned image (7000 points), ensuring high internal statistical consistency. The presented results reflect spatial variability within the sample and do not account for inter-sample repeatability. Comprehensive uncertainty analysis, including confidence intervals and intersample variability assessments, requires the preparation and analysis of multiple independent dosimeters and may constitute a separate research topic for future optimisation studies of the TTC-Pluronic F-127 system.

### 3.3. Resolution of the TTC-Pluronic F-127 Dosimeter with a Flatbed Scanner Readout

**Figure 9:**
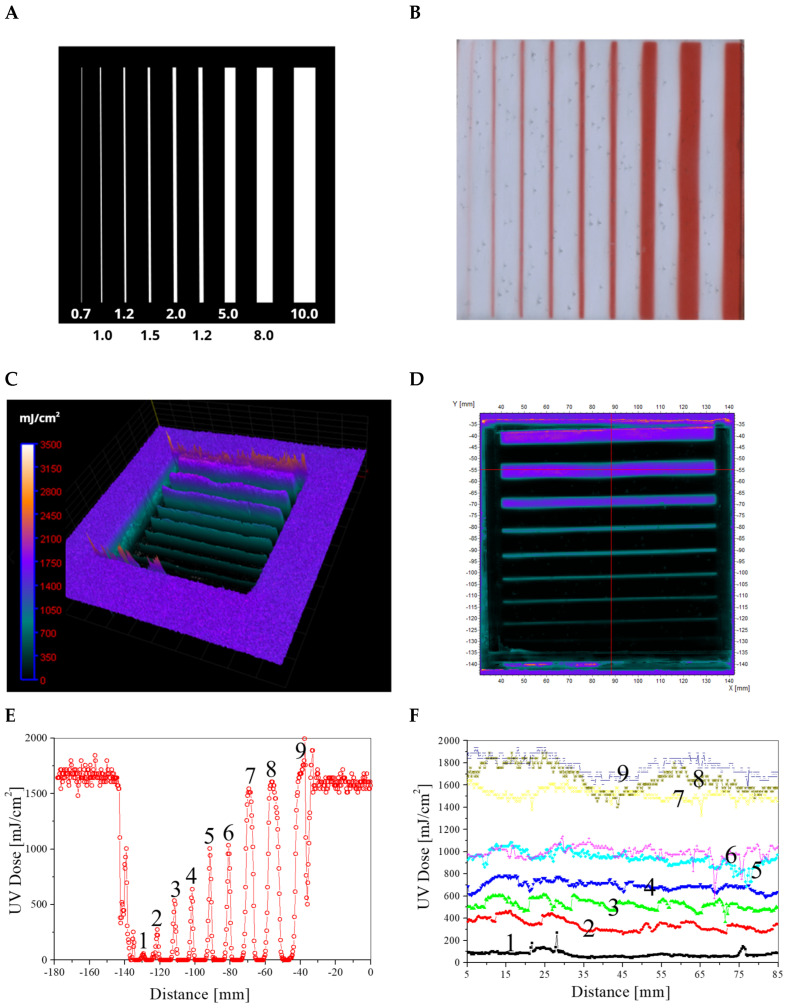
Dose distribution as measured by the 2D TTC-Pluronic F-127 dosimeter irradiated with UVA radiation through the template with stripe-like areas. (**A**) irradiation pattern template with 0.7–10 mm thick lines. (**B**) scanned image of the TTC-Pluronic F-127 sample after irradiation with a dose of 2000 mJ/cm^2^. (**C**) 3D view of the 2D dose distribution; the colour bar corresponds to the dose range [mJ/cm^2^]. (**D**) 2D view of the dose distribution with the solid red lines along the second stripe from the top and across all stripes to illustrate the position for the profile along Y-axis in (**E**) and the profiles along X-axis in (**F**). The thickness of the lines is 0.7, 1.0, 1.25, 1.5, 2.0, 2.25, 5.0, 8.0, and 10 mm from bottom to top in (**C**,**D**). Numbers from 1 to 9 (**E**,**F**) correspond to the lines, where 1 is 0.7 mm and 9 is 10 mm thick.

**Figure 10:**
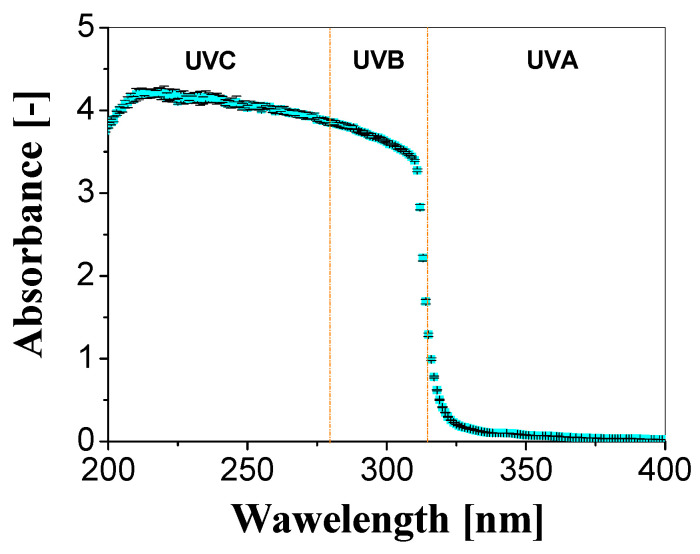
The absorbance measurements in the range of 190–700 nm for the CtF film used for irradiation pattern prints. The red dashed lines are intended to guide the eye and differentiate between the UV subranges.

The precision of 2D gel dosimeters depends on factors including their preparation method, irradiation conditions, and reading using scanning or spectrophotometric measurements. In the case of TTC-Pluronic-127 dosimeters, maintaining chemical stability and consistent gel geometry is crucial. During measurements using a flatbed scanner, scanning resolution and subsequent image filtering in the dedicated software have a significant impact. It is important to remember that flat gel samples are susceptible to damage due to handling during measurements. Accidental pressure on their surface should be avoided, as this can lead to local changes in sample thickness, which can alter the colour distribution and distort the obtained data. Evaluating the spatial resolution of a dosimeter, understood as its ability to reproduce geometric features of an irradiation pattern, is a key benchmark for determining the suitability of such systems, particularly when examining complex areas irradiated with steep dose gradients. In this work, spatial resolution is defined operationally as the minimum distinguishable feature size based on the ability to resolve line structures and their corresponding dose profiles, providing a practical, semi-quantitative estimate under the applied experimental conditions. To analyse this aspect, a pattern was printed on CtF film simulating a multi-slit mask with lines ranging from 0.7 to 10 mm thick (Figure 9A). The sample was then irradiated with a dose of 2000 mJ/cm^2^ and scanned. The resulting image (Figure 9B) was processed using polyGeVero^®^-CT software according to the procedure described in Section 2.3. After assigning the calibration equation, it was possible to obtain a 2D dose distribution graph, which is presented in Figure 9C,D. The UVA dose change as a function of distance on the sample was determined along with the uniformity profile in areas with thicknesses of 0.7, 1.0, 1.25, 1.5, 2.0, 2.25, 5.0, 8.0, and 10 mm (Figure 9E,F). The analysis indicated that the TTC-Pluronic F-127 system changed colour after irradiation and could measure colour changes across a wide range of tested line thicknesses, even the narrower line of 0.7 mm is recognisable on an image. The narrower the line, the lower recorded dose was observed (Figure 9E,F). This effect may result from the optical properties of the template itself, which was printed on CtF film, and the overlapping of the two foils during the irradiation process. While the CtF film itself is highly transparent and, as described in the previous chapter, does not significantly reduce UV absorbance, the high-contrast printed pattern can have a scattering effect. Although visually opaque, the print used in CtF technology has a specific optical density, which can also cause uneven UV radiation distribution on such a surface. Unlike a physical aperture, a printed line on CtF film has a finite thickness and microscopic surface roughness. When UV radiation passes through the edges of these printed segments, phenomena such as diffraction and radiation scattering occur. These effects contribute to the formation of penumbra at the boundaries of the irradiated zones. Therefore, in the case of narrow lines, the radiation dose may be attenuated compared to wider areas. Additionally, it should be emphasised that the CtF film template adhered to the dosimeter sample surface during irradiation by means of a two-surface contact, where a loss of radiation absorption could have occurred due to the contact between the air-CtF film and the gel-PET foil, resulting in a reduced dose absorbed by the dosimeter. Problems related to the physicochemical structure of CtF for screen printing are known and well described in the literature [31,32,33]. Errors in the irradiation process using CtF films are caused by (i) interference oscillation phenomena, (ii) thickness inhomogeneity, and (iii) light scattering and loss of sharpness. Although CtF films appear smooth and highly transparent, they often lack uniform thickness. Varying film thickness can affect the transmission of radiation from typical UV lamps at 365 nm and 405 nm wavelengths [31]. Furthermore, local film densification and CtF film structure affect variations in UVA scattering and significantly influence its transmission and absorption [32,33]. Given that the UV chamber used in the study employed UVA lamps with a maximum wavelength of 369 nm, it was necessary to verify whether the phenomena described above could significantly impact the difference between the emitted dose by irradiator and absorbed dose by the TTC-Pluronic F-127 dosimeter. The conducted studies show that in the 315–400 nm range, the light absorbance of the CtF film sample is very low, and the unprinted surface alone should not significantly reduce the absorbed UVA dose (Figure 10). It should be noted that, in addition to optical effects associated with the use of CtF films, changes in gel thickness can also affect the measured dose distribution, for example, through changes in optical path length and internal light scattering within the hydrogel matrix, potentially leading to differences in the recorded colour intensity, resolution, and dose response. In this study, all measurements were performed using samples with a constant thickness, defined by the PMMA container, so this effect was not investigated. Systematic evaluation of the effect of gel thickness, including the definition of acceptable thickness ranges for accurate and stable dose measurements, remains an important topic for future research, particularly in applications involving variable geometry or the use of different sample containers.

**Figure 11:**
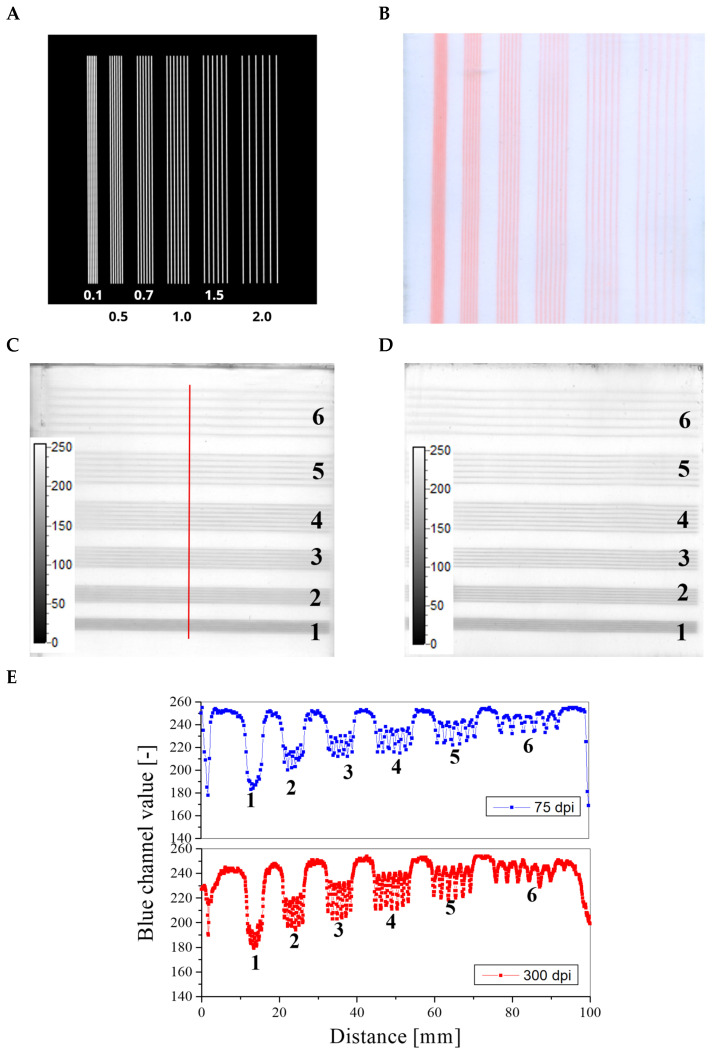
Irradiation line pattern (**A**) and scanned image of the TTC-Pluronic F-127 sample after irradiation with UVA (500 mJ/cm^2^) (**B**). Blue channel (RGB) image (**C**,**D**), and profiles (**E**) across all the lines as indicated in C with the red solid line. The distances between the 1.0 mm thick lines are as follows: 1—0.1; 2—0.5; 3—0.7; 4—1.0; 5—1.5; and 6—2.0 mm. The sample was scanned with a flat-bed scanner: 75 dpi (**C**) and 300 dpi (**D**).

In the next step, we tested the impact of spacing between 1.0 mm thick lines on their proper readout by the TTC-Pluronic F-127 dosimeter. For this purpose, a CtF film irradiation template was prepared (Figure 11A), with line spacings of 0.1, 0.5, 0.7, 1.0, 1.5, and 2.0 mm, respectively. The irradiated sample (Figure 11B) was scanned using a flatbed scanner, and the obtained images were further processed (Figure 11C–E). For both 75 and 300 dpi scanning resolution, lines spaced 0.1 mm apart are difficult to distinguish in the graphs. For lines spaced 0.5–2.0 mm apart, distinct changes in the intensity of the blue channel are visible at both resolutions. In summary, the TTC-Pluronic F-127 dosimeter demonstrated the ability to precisely record dose in 2D. Tests using CtF film radiation templates confirmed that the dosimeter effectively distinguished lines 1.0 mm thick while maintaining sharp edges and minimal blurring, indicating the absence of significant formazan diffusion within the matrix. Although a decrease in the detected dose was observed in lines narrower than 5 mm, this was primarily due to the optical limitations of the CtF film template and not to any defects in the dosimeter itself. In line spacing resolution tests, the dosimeter demonstrated a resolution limit of above 0.1 mm. These results confirm that the presented experiments evaluate the spatial resolution of the dosimeter, i.e., its ability to reproduce fine structural details of the irradiation pattern. A more rigorous characterisation using imaging system metrics such as modulation transfer function was not performed and remains a subject for future work.

**Figure 12:**
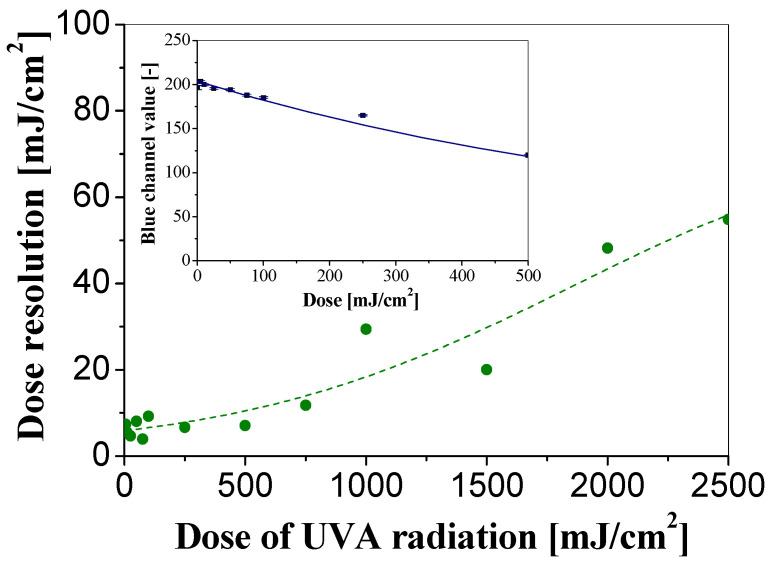
Dose resolution of the TTC-Pluronic F-127 dosimeter. Insert: fragment of the calibration dependence of the dosimeter in the dose range of 0–500 mJ/cm^2^.

Independently of spatial resolution, the dose resolution (*D^p^_Δ_*), defined as the minimum detectable difference in absorbed dose [34], was determined based on statistical analysis of the scanned dosimeter images, according to the methodology described in [32]. Figure 12 presents the resolution of the developed TTC-Pluronic F-127 dosimeter, which was calculated based on the formula: $D_{∆}^{p}=k_{p}\cdot \sqrt{2}\cdot \sigma_{D}$, where $k_{p}$ is the quantile coefficient of the normal distribution (95% confidence level) and $\sigma_{D}$ is the standard deviation on dose. The obtained results indicate that the resolution of the dosimeter deteriorates with increasing UVA radiation dose. In the measurement range up to 100 mJ/cm^2^, a high measurement uncertainty is visible, resulting from problems with precise dose emission, which were previously described in Section 3.1. Above a dose of 1000 mJ/cm^2^, the developing dosimeter reaches maximum colour saturation after irradiation (Figure 8), and the resolution decreases.

The dose resolution was determined based on a single physical dosimeter sample. However, statistical analysis was performed using data points obtained from a scanned image (7000 points). The parameter *σ_D_* represents the standard deviation of the dose value calculated based on the distribution of pixels in selected regions of interest corresponding to uniformly irradiated areas. Therefore, the uncertainty reflects spatial variability within the dosimeter, not inter-sample variability. The calculations assumed a normal distribution of dose values within the ROIs, and the coefficient *k_p_* corresponds to the 95% confidence level. The factor $\sqrt{2}$ accounts for error propagation when comparing two dose levels, which is consistent with standard methods used in gel dosimetry. It should be noted that this approach does not account for inter-sample reproducibility, and further studies including replicate measurements are required for full statistical validation of the dose resolution.

### 3.4. Inhomogeneous Dose Distribution

**Figure 13:**
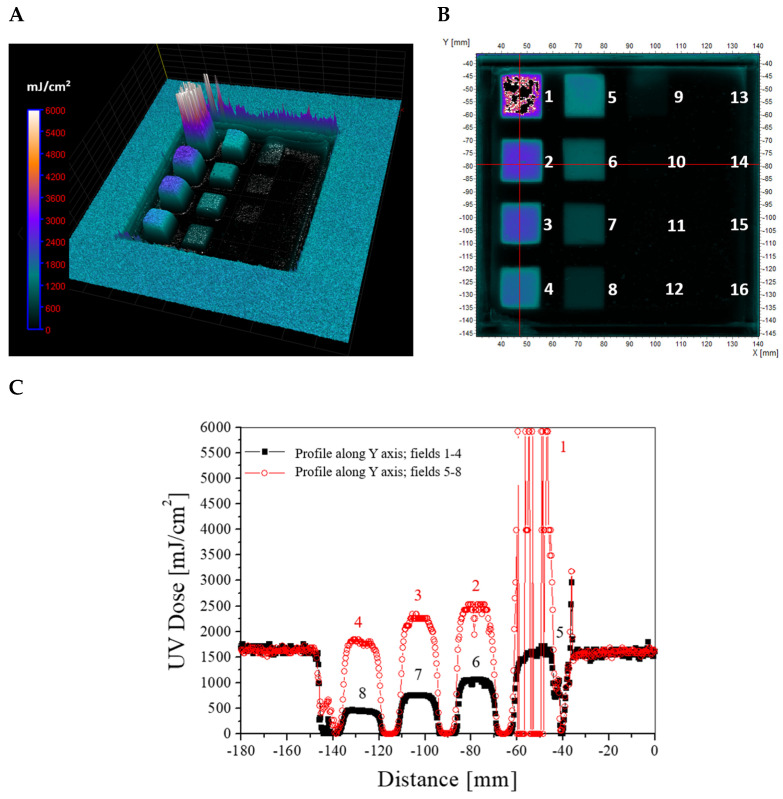
Dose distribution as measured by the 2D TTC-Pluronic F-127 dosimeter irradiated with UVA radiation through the template with square areas. (**A**) 3D view of the 2D dose distribution; the colour bar corresponds to the dose range [mJ/cm^2^]. (**B**) The 2D dose distribution map with the red solid lines indicating positions of the profiles along *Y*-axis presented in (**C**) (corresponds to fields 1–8).

**Figure 14:**
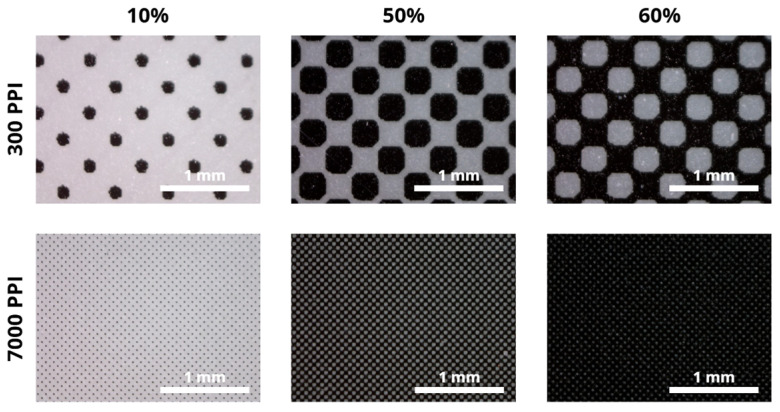
Microscopic comparison of prints on CtF film with 10%, 50%, and 60% black coverage for 300 and 7000 ppi screen raster rulings. Images were taken with a Smart 5MP PRO digital microscope (2592 × 1944 pixel image, 5MP resolution, Delta Optical, Minsk Mazowiecki, Poland).

The TTC-Pluronic F-127 dosimeter was subjected to inhomogeneous irradiation. As before, templates printed on CtF film were used for irradiation. In the first stage, a template was used divided into 16 fields measuring 18 × 18 mm^2^ each of which was darkened by 6.25% compared to the previous one, ranging from 0% to 93.75% of black print. The dosimeter was irradiated with a dose of 3000 mJ/cm^2^, scanned, and further analysed. Similar to the analysis described in Section 3.2, after assigning the calibration equation, dose distribution maps and profiles were prepared and presented in Figure 13. The obtained data indicate that a CtF film opacity above 50% prevents the developed dosimeter from recording UV radiation. Therefore, with inhomogeneous irradiation, the type of irradiation patterns used is crucial. Problems with UV radiation penetration through the printed surface of CtF film have already been partially described in Section 3.3. Although at first glance films printed in different shades appear uniformly covered in black, the print consists of microscopic dots specifically distributed on the surface of the transparent film. Figure 14 presents a microscopic view of two printed patterns with different screen raster rulings, for 300 and 7000 ppi (points per inch), with the same black opacity levels for 10%, 50%, and 60%, respectively. Analysing the densitometric change in these test fields, increasing the raster dot ruling while maintaining a constant surface coverage can significantly affect the amount of UV radiation passing through the CtF film. Undoubtedly, changes in the scattering, transmission, and diffraction of radiation occur on the surface of such a template, which in practice translates into the dose absorbed by the developed dosimeter. Additionally, dose measurements were performed using a Sentry ST510 UV meter for the CtF samples. To ensure uniform irradiation conditions, as described in Section 3.1, measurements were taken in the central field of the crosslinker chamber. The samples were irradiated with a dose of 100 mJ/cm^2^, and the results are presented in Table 4. The results indicate that the dose decreases depending on the level of black overprinting on the CtF film. For the unprinted film, the dose is approximately 20% lower than the radiated dose; for the films with 10%, 50%, and 60% overprinting, the dose decreases by 28%, 64%, and 65% for the 300 PPI screen raster rulings, and by 29%, 67%, and 83% for the 7000 PPI screen raster rulings, respectively.

**Figure 15:**
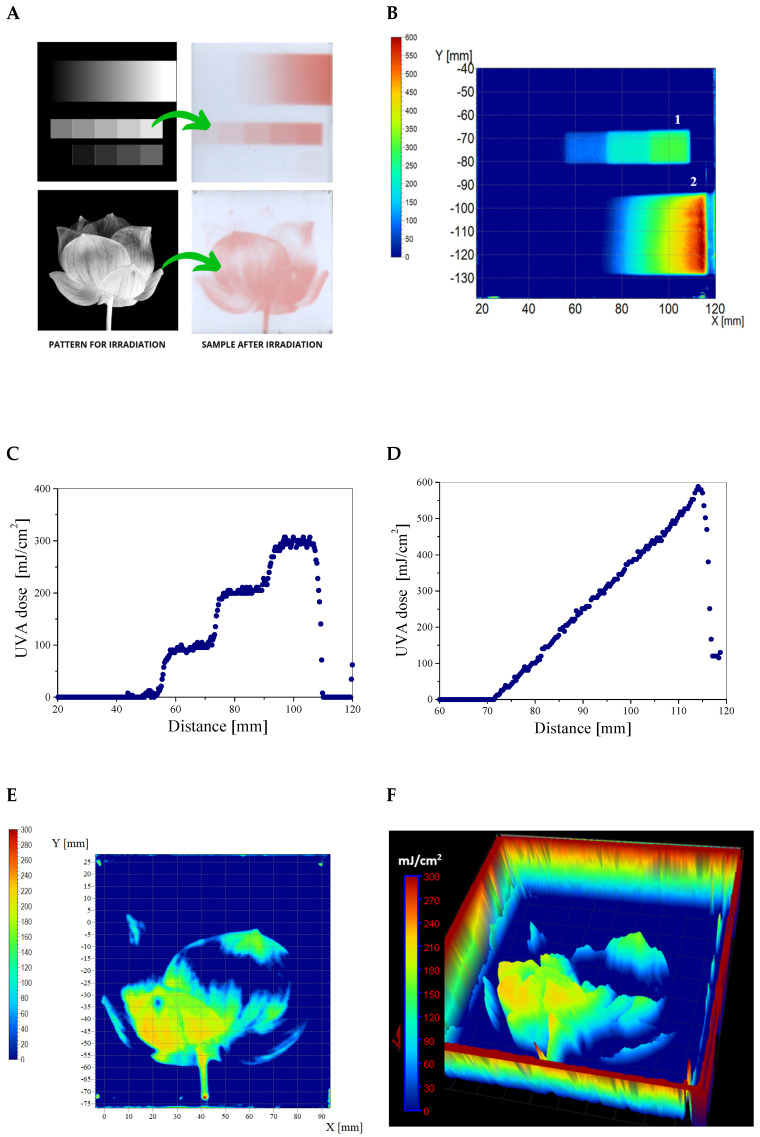
Dose distribution for TTC-Pluronic F-127 samples irradiated with a particular pattern. (**A**) a design imitating dose gradient and a flower. Samples irradiated with 600 mJ/cm^2^ UVA. (**B**) 2D dose distribution of design imitating dose gradient. (**C**,**D**) a profile along the stepped gradient dose distribution (1) and the uniform gradient dose distribution (2) corresponds to (**B**). (**E**,**F**) 3D visualizations of 2D dose distributions of irradiated TTC-Pluronic F-127 sample with flower pattern.

Therefore, when using simple templates printed on CtF film for irradiation, calibration for each field with a known black coverage would be necessary. This problem becomes more complicated when templates with non-uniform shapes and black coverage are used for irradiation, as it is impossible to determine the dose reduction at each point of the template. This is important from the perspective of real irradiation processes. Therefore, non-homogeneous dose distribution measurements were performed using a template with uneven black printing in the form of a changing stripe imitating the dose gradient and a flower shape, which are presented in Figure 15A. As shown in Figure 15B, the dosimeter can measure radiation doses passing through black-printed areas in the range of 0–40%, which is also confirmed by the UV dose vs. distance graphs (Figure 15C,D). Importantly, no interference was observed in the gradient dose increment simulation, which also demonstrates the high homogeneity of the raster dot distribution in the printed area of the CtF film. In the case of the flower pattern (Figure 15E,F), the general outline of the irradiated shape is visible, and doses in the range of up to approximately 250 mJ/cm^2^ were recorded, confirming the previously described problems with using CtF film patterns for UV irradiation. It should be emphasized that the observed dose variations are due to two factors: the intrinsic response of the TTC-Pluronic F-127 dosimeter and the optical effects introduced by the CtF film, including scattering, diffraction, and local thickness variations. Full quantitative separation of these factors would require dedicated optical characterization of the CtF film, including measurements of UV transmission through the CtF film with controlled and independently variable parameters (e.g., screen ruling, opacity, and thickness); comparison with irradiation conditions without intermediate templates; and potentially modeling or optical characterization, e.g., using spectrophotometric analysis or angle scattering. Due to material and technological limitations, this was beyond the scope of the presented study.

## 4. Conclusions

The research demonstrated that Pluronic F-127 hydrogel with 2,3,5-triphenyltetrazolium chloride is an effective and precise tool for two-dimensional assessment of UVA radiation dose distribution. The polymer matrix used is non-toxic and can be easily formed into flat layers, making it a safe alternative to traditional dosimetry systems used in UV radiation work. Furthermore, the developed dosimeter is characterised by high transparency and mechanical stability, which, combined with the radiochromic properties of TTC, enables the generation of accurate irradiation maps through analysis of colour TTC formazan formation. A key conclusion from the conducted experiments is the confirmation of significant field heterogeneity in standard irradiation chambers, where deviations from the target dose reached up to 40%, clearly indicating the need for 2D dosimetry in the validation processes of such devices. The obtained results confirmed that precise 2D dosimetry is essential to eliminate systematic errors in UV radiation studies. Furthermore, the demonstrated effect of PET and CtF films on beam attenuation (20–21%) is an important correction parameter that should be considered in measurement procedures, both in the calibration process and in the interpretation of dosimetric results. The TTC-Pluronic F-127 dosimeter, combined with scanning methods, digital image processing and the generation of dose distribution maps, demonstrates high application potential, for instance, in the quality control of medical and cosmetic lamps and in research on the photodegradation of materials. Compared to previously described UV dosimeters based on TTC and Pluronic F-127, which were primarily limited to point measurements, simple geometries, or qualitative response analysis, the system presented in this study enables quantitative two-dimensional mapping of UVA radiation dose distributions using image analysis. In the broader context of UV dosimetry, the proposed TTC–Pluronic F-127 system can be positioned as a solution combining direct spatial dose visualisation with a relatively simple and inexpensive readout method. In the studied dose range (0–5000 mJ/cm^2^), the dosimeter is characterised by high sensitivity and sufficient resolution to reproduce complex irradiation patterns under real-world conditions. Unlike conventional methods based on local or integrated measurements, the presented system provides dose information with resolution, which is particularly important when dosimeters are used to measure inhomogeneous radiation fields. At the same time, certain limitations must be considered, such as the time-dependent structural instability of the hydrogel matrix, sensitivity to optical artefacts, and the need for controlled sample preparation and use. This topic is not fully explored and requires further optimisation, especially in the context of investigating the influence of gel thickness and mechanical deformation on the dosimetry response, long-term stability, and reproducibility improvements.

## Figures and Tables

**Table 1:** Measurements of UVA radiation intensity and dose measurements in the CL-1000 crosslinker chamber, with a Sentry ST510 UV meter, and 2D dose distribution map with analysis of the crosslinker chamber homogeneity at a dose set at 100 mJ/cm^2^. Field numbers are indicated in Figure 1.

UVA set dose: 100 mJ/cm^2^	**Field No.**	**Medium Intensity [mW/cm^2^]**	**Measured Dose [mJ/cm^2^]**	**Deviation from the Set Dose** **[%]**	**Coefficient of Variation (CV) [%]**
	1	3.44 ± 0.30	61.86	38.14	6.38
	2	3.70 ± 0.21	66.54	33.46	2.89
	3	3.88 ± 0.19	69.78	30.22	0.39
	4	4.05 ± 0.19	72.84	27.16	0.14
	5	5.19 ± 0.25	93.48	6.52	0.11
	6	5.12 ± 0.24	92.10	7.90	0.30
	7	5.12 ± 0.24	92.10	7.90	0.41
	8	5.13 ± 0.24	92.28	7.72	0.49
	9	5.12 ± 0.24	92.10	7.90	0.63
	10	4.88 ± 0.23	87.84	12.16	0.20
	11	4.98 ± 0.23	89.70	10.30	0.23
	12	4.86 ± 0.23	87.54	12.46	0.31
	13	3.63 ± 0.17	65.28	34.72	0.32
	14	3.25 ± 0.16	58.44	41.56	0.36
	15	3.34 ± 0.16	60.06	39.94	0.17
	16	3.54 ± 0.17	63.78	36.22	0.16
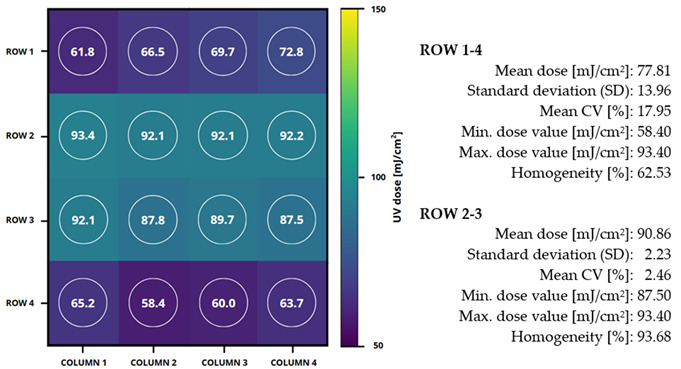					

**Table 2:** Measurements of UVA radiation intensity and dose measurements in the CL-1000 crosslinker chamber, with a Sentry ST510 UV meter, and 2D dose distribution map with analysis of the crosslinker chamber homogeneity at a dose set at 500 mJ/cm^2^. Field numbers are indicated in Figure 1.

UVA set dose: 500 mJ/cm^2^	**Field No.**	**Medium Intensity [mW/cm^2^]**	**Measured Dose [mJ/cm^2^]**	**Deviation from the Set Dose** **[%]**	**Coefficient of Variation (CV) [%]**
	1	3.90 ± 0.19	343.20	31.36	0.26
	2	4.04 ± 0.20	355.52	28.90	0.50
	3	4.06 ± 0.20	356.99	28.60	0.14
	4	4.08 ± 0.20	358.75	28.25	0.37
	5	5.08 ± 0.26	447.04	10.59	1.38
	6	4.94 ± 0.25	434.43	13.11	1.32
	7	4.84 ± 0.24	426.21	14.76	1.06
	8	4.87 ± 0.24	428.85	14.23	0.63
	9	4.79 ± 0.24	421.81	15.64	1.07
	10	4.65 ± 0.23	409.20	18.16	0.37
	11	4.62 ± 0.24	406.56	18.69	1.35
	12	4.46 ± 0.22	392.19	21.56	0.93
	13	3.49 ± 0.18	307.12	38.58	1.03
	14	3.28 ± 0.17	288.64	42.27	1.61
	15	3.01 ± 0.15	264.59	47.08	0.69
	16	3.55 ± 0.17	312.11	37.58	10.83
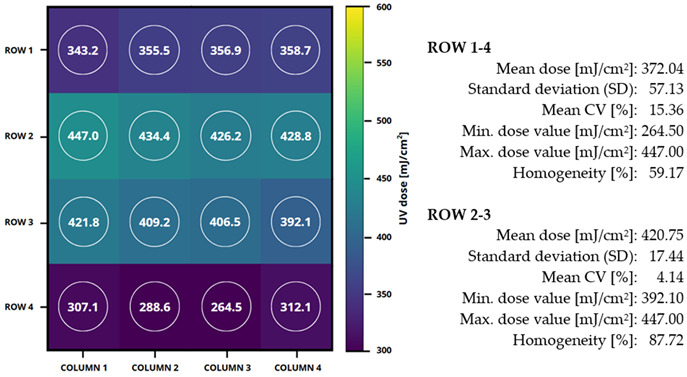					

**Table 3:** Measurements of UVA radiation intensity and dose measurements in the CL-1000 crosslinker chamber, with a Sentry ST510 UV meter, and 2D dose distribution map with analysis of the crosslinker chamber homogeneity at a dose set at 1000 mJ/cm^2^. Field numbers are indicated in Figure 1.

UVA set dose: 1000 mJ/cm^2^	**Field No.**	**Medium Intensity [mW/cm^2^]**	**Measured Dose [mJ/cm^2^]**	**Deviation from the Set Dose** **[%]**	**Coefficient of Variation (CV) [%]**
	1	3.70 ± 0.18	722.15	27.79	0.56
	2	3.70 ± 0.18	721.50	27.85	0.72
	3	3.75 ± 0.19	730.60	26.94	1.34
	4	3.71 ± 0.19	724.10	27.59	1.91
	5	4.63 ± 0.23	903.50	9.65	0.87
	6	4.67 ± 0.22	910.65	8.94	0.57
	7	4.64 ± 0.23	905.45	9.46	1.11
	8	4.58 ± 0.23	893.75	10.63	1.67
	9	4.54 ± 0.22	885.95	11.41	1.21
	10	4.54 ± 0.23	884.65	11.54	1.88
	11	4.49 ± 0.24	874.90	12.51	2.24
	12	4.34 ± 0.21	845.65	15.44	0.81
	13	3.29 ± 0.17	642.20	35.78	1.73
	14	2.81 ± 0.14	548.60	45.14	1.48
	15	2.73 ± 0.14	533.00	46.70	1.73
	16	2.89 ± 0.14	563.55	43.65	1.25
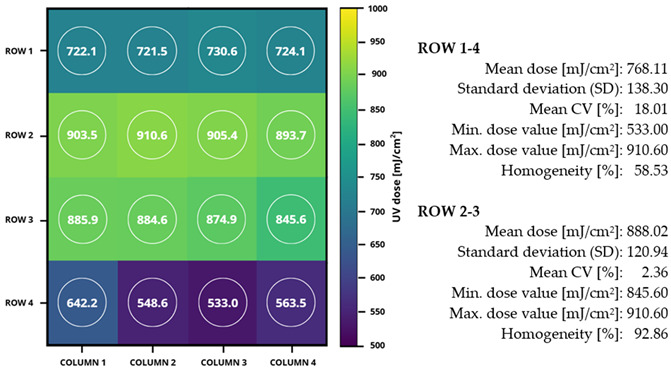					

**Table 4:** Changes in UV doses read by TTC-Pluronic F-127 irradiated to 100 mJ/cm^2^ depending on the type of CtF film used as a template for irradiation.

CtF Type	None	Clear CtF	300 PPI			7000 PPI		
Black coverage	-	-	10%	50%	60%	10%	50%	60%
Dose [mJ/cm^2^]	91.22	72.98	65.52	32.04	31.32	64.08	29.34	14.94

## Data Availability

The original contributions presented in the study are included in the article, further inquiries can be directed to the corresponding authors.
